# Supplementary material for: Beta variant COVID-19 protein booster vaccine elicits durable cross-neutralization against SARS-CoV-2 variants in non-human primates
Source: Nat Commun. 2023 Mar 10;14:1309. doi: 10.1038/s41467-023-36908-z (PMC9998256; doi:10.1038/s41467-023-36908-z)
Supplement: Supplementary file 1 — Supplementary Information [file 41467_2023_36908_MOESM1_ESM.pdf]

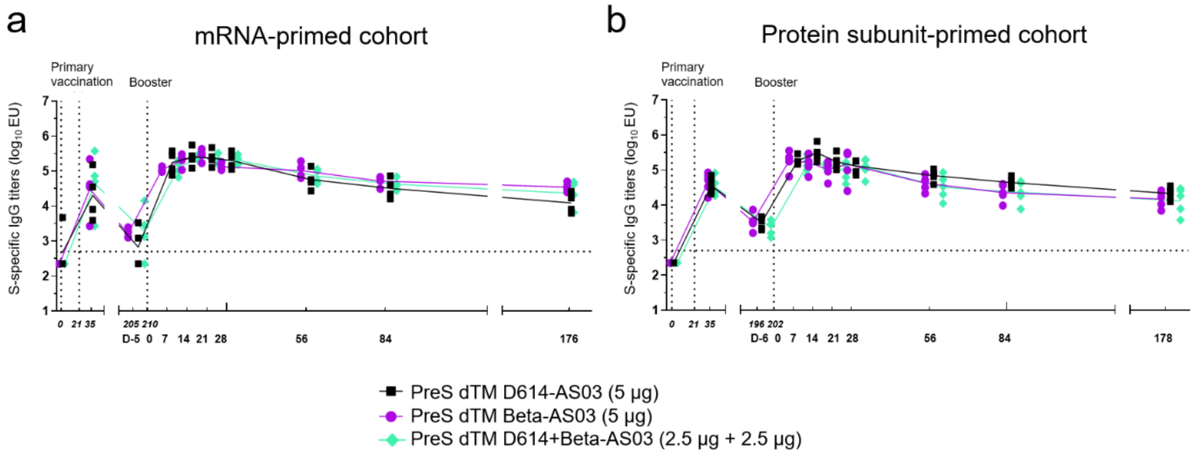

Supplementary Fig. 1 Spike-specific IgG ELISA. Spike-specific binding antibody responses were assessed by ELISA in macaques before and after the primary vaccination with **a** mRNA vaccine candidate (n=4) or **b** protein-subunit vaccine candidate (n=5) and up to day 176 or 178 following booster with CoV2 preS dTM-AS03. Individual macaque data are shown. Connecting lines indicate mean responses and horizontal dotted lines the limits of quantification of the assay.
