# Supplementary material for: Beta variant COVID-19 protein booster vaccine elicits durable cross-neutralization against SARS-CoV-2 variants in non-human primates
Source: Nat Commun. 2023 Mar 10;14:1309. doi: 10.1038/s41467-023-36908-z (PMC9998256; doi:10.1038/s41467-023-36908-z)
Supplement: Supplementary file 3 — Reporting Summary [file 41467_2023_36908_MOESM3_ESM.pdf]

nature portfolio

Corresponding author(s): V. Lecouturier

Last updated by author(s): Jan 17, 2023

Reporting Summary

Nature Portfolio wishes to improve the reproducibility of the work that we publish. This form provides structure for consistency and transparency in reporting. For further information on Nature Portfolio policies, see our Editorial Policies and the Editorial Policy Checklist.

Statistics

For all statistical analyses, confirm that the following items are present in the figure legend, table legend, main text, or Methods section.

n/a	Confirmed	
☐	☒	The exact sample size (n) for each experimental group/condition, given as a discrete number and unit of measurement
☐	☒	A statement on whether measurements were taken from distinct samples or whether the same sample was measured repeatedly
☐	☒	The statistical test(s) used AND whether they are one- or two-sided<br>*Only common tests should be described solely by name; describe more complex techniques in the Methods section.*
☒	☐	A description of all covariates tested
☐	☒	A description of any assumptions or corrections, such as tests of normality and adjustment for multiple comparisons
☐	☒	A full description of the statistical parameters including central tendency (e.g. means) or other basic estimates (e.g. regression coefficient) AND variation (e.g. standard deviation) or associated estimates of uncertainty (e.g. confidence intervals)
☐	☒	For null hypothesis testing, the test statistic (e.g. F, t, r) with confidence intervals, effect sizes, degrees of freedom and P value noted<br>*Give P values as exact values whenever suitable.*
☒	☐	For Bayesian analysis, information on the choice of priors and Markov chain Monte Carlo settings
☒	☐	For hierarchical and complex designs, identification of the appropriate level for tests and full reporting of outcomes
☒	☐	Estimates of effect sizes (e.g. Cohen's d, Pearson's r), indicating how they were calculated

Our web collection on statistics for biologists contains articles on many of the points above.

Software and code

Policy information about availability of computer code

Data collection	The data collection and titer calculation were performed using a proprietary software, Sanofi Universal Exporter 2.1. The application takes raw data file from the instruments and transfer them automatically to predefined protocols on the Softmax Pro 6.5.1 GxP software where the calculations are performed, and analyzed according to pre-defined validation criteria.
Data analysis	The analyses were performed on SEG SAS v9.4®

For manuscripts utilizing custom algorithms or software that are central to the research but not yet described in published literature, software must be made available to editors and reviewers. We strongly encourage code deposition in a community repository (e.g. GitHub). See the Nature Portfolio guidelines for submitting code & software for further information.

Data

Policy information about availability of data

All manuscripts must include a data availability statement. This statement should provide the following information, where applicable:

- Accession codes, unique identifiers, or web links for publicly available datasets
- A description of any restrictions on data availability
- For clinical datasets or third party data, please ensure that the statement adheres to our policy

Accession codes, and web links for publicly available datasets are provided in the manuscript. The source data generated in this study are included in this paper and the supplementary information

Field-specific reporting

Please select the one below that is the best fit for your research. If you are not sure, read the appropriate sections before making your selection.

☒ Life sciences ☐ Behavioural & social sciences ☐ Ecological, evolutionary & environmental sciences

For a reference copy of the document with all sections, see nature.com/documents/nr-reporting-summary-flat.pdf

Life sciences study design

All studies must disclose on these points even when the disclosure is negative.

Sample size	No sample size calculation was done as the number of animals was limited by the availability of primed animals from 2 prior studies.
Data exclusions	No data were excluded from the analysis
Replication	All assays used for the analytical measures (ELISA and viral neutralization) included internal control to ensure the reproducibility of the measurements. Repeat measures were performed on subsets of sample to control for the reproducibility
Randomization	Due to the multiple baseline factors to be considered, randomization of the animal was not performed. However, the characteristics at baseline (sex, age, weight) were balanced in order to have comparable groups. The pseudovirus neutralizing titers after priming immunization were also taken into account as well as the vaccine used for the primary immunization.
Blinding	No blinding was used as sample blinding is not typically used for non-clinical studies, and would have introduced some logistical complexity at different steps (sample collection, storage and analysis).

Reporting for specific materials, systems and methods

We require information from authors about some types of materials, experimental systems and methods used in many studies. Here, indicate whether each material, system or method listed is relevant to your study. If you are not sure if a list item applies to your research, read the appropriate section before selecting a response.

Materials & experimental systems

n/a	Involved in the study	
☐	☒	Antibodies
☐	☒	Eukaryotic cell lines
☒	☐	Palaeontology and archaeology
☐	☒	Animals and other organisms
☒	☐	Human research participants
☒	☐	Clinical data
☒	☐	Dual use research of concern

Methods

n/a	Involved in the study	
☒	☐	ChIP-seq
☒	☐	Flow cytometry
☒	☐	MRI-based neuroimaging

Antibodies

Antibodies used	Secondary goat anti-human IgG (Jackson Immuno Research, CAT# 109-036-098)
Validation	No validation was performed. Antibodies were all from commercial source, and either human polyclonal antibodies used as comparators or secondary antibodies.

Eukaryotic cell lines

Policy information about cell lines

Cell line source(s)	hsACE2-expressing HEK293T cells were obtained from Integral Molecular (catalog No. C-HA102)
Authentication	No authentication was performed after purchase from Integral Molecular.
Mycoplasma contamination	The cell line was not tested for mycoplasma contamination after receipt.
Commonly misidentified lines (See ICLAC register)	No commonly misidentified cell lines were used.

Animals and other organisms

Policy information about studies involving animals; ARRIVE guidelines recommended for reporting animal research

Laboratory animals	Adult male and female Mauritius cynomolgus macaques (Macaca fascicularis) aged 4-10 years, and adult male Indian rhesus macaques (Macaca mulatta) aged 4-7 years were used.
Wild animals	The study did not involve wild animals, all animals were from colonies established at New Iberia Research Center.
Field-collected samples	The study did not involve samples collected from field.
Ethics oversight	The study was approved by the Institutional Animal Care and Use Committee (IACUC) from the University of Louisiana at Lafayette New Iberia Research Center (IACUC number 2020-8733-013)

Note that full information on the approval of the study protocol must also be provided in the manuscript.
